# The *GC2* haplotype of the vitamin D binding protein is a risk factor for a low plasma 25-hydroxyvitamin D concentration in a Han Chinese population

**DOI:** 10.1186/s12986-019-0332-0

**Published:** 2019-01-14

**Authors:** Ji-Chang Zhou, Yumei Zhu, Chunmei Gong, Xiongshun Liang, Xiaoying Zhou, Yuanfei Xu, Deliang Lyu, Junluan Mo, Jian Xu, Jinping Song, Xiaoling Che, Shiqiang Sun, Changhua Huang, Xiao-Li Liu

**Affiliations:** 1 Shenzhen Centre for Chronic Disease Control, 2021 Buxin Road, Luohu District, Shenzhen, 518020 Guangdong China; 2 School of Public Health (Shenzhen), Sun Yat-sen University, Shenzhen, 518100 Guangdong China; 3 Shenzhen Qilinshan Sanatorium, Shenzhen, 518055 Guangdong China

**Keywords:** Vitamin D binding protein, Single nucleotide polymorphism, Circulating 25-hydroxyvitamin D, Glucose, Lipids, Han Chinese

## Abstract

**Background:**

The *GC* haplotype of the vitamin D binding protein (encoded by the *GC* gene) might be a risk factor to the vitamin D (VD) nutritional status for many populations, while evidences from the Chinese Han population are sparse. We test the association between vitamin D binding protein genotypes and VD status as well as the metabolic parameters of glucose and lipids in a Han Chinese population.

**Methods:**

In a cross-sectional study conducted at a health examination centre (registered in ClinicalTrials.gov as QLS2013), 2641 adults were included and grouped according to their plasma 25-hydroxyvitamin D (25OHD) concentrations as VD deficient (VDD), insufficient (VDI), or sufficient (VDS). The rs7041 and rs4588 genotypes were analysed with a molecular beacon-based qPCR method using blood samples.

**Results:**

Plasma 25OHD concentrations were lower in the *GC*2/2, rs7041T/T, and rs4588A/A genotypes than the *GC*1f/1s, rs7041G/T, and rs4588C/C genotypes (*P* <  0.05). After adjusting for confounders, the *GC*2 haplotype increased the risk of low VD status (*P* <  0.05) in both genders. More genotypic models revealed the negative contributions of rs4588A than rs7041T to low VD status (*P* <  0.05). The combined rates of VDD and VDI were 80.2% in males and 86.1% in females. Compared with VDI, VDS, or both, VDD showed higher plasma concentrations of fasting blood glucose, high-density lipoprotein cholesterol, low-density lipoprotein cholesterol, and triglycerides in males (*P* <  0.05); however, no significant differences were found with regard to these parameters between the subgroups defined by the *GC* genotypes (*P* > 0.05).

**Conclusions:**

In a Han Chinese population, the *GC2* haplotype or more exactly rs4588A is a risk factor for low VD status but is not associated with glucose and lipid metabolic disorders, which are inversely correlated with the circulating 25OHD concentration in males.

**Trial registration:**

The study was retrospectively registered in January 2018 as NCT03406234 in the ClinicalTrials.gov online system.

**Electronic supplementary material:**

The online version of this article (10.1186/s12986-019-0332-0) contains supplementary material, which is available to authorized users.

## Background

The high prevalence of vitamin D (VD) malnutrition (deficiency and insufficiency) has been recognized as a global public health problem, even in areas with a significant amount of sunshine [1]. Furthermore, this condition might be related to the high incidences of many chronic diseases, not only that of bone health [2–4]. Specifically, the metabolic disorders associated with glucose and lipids might be affected by lower VD nutritional status [5–11]. These conditions have attracted a growing amount of attention because they are pilot phenotypes for metabolic syndrome, diabetes, dyslipidaemia, and other conditions. The concentration of plasma/serum 25-hydroxyvitamin D (25OHD) is commonly accepted as an indicator of VD nutritional status [2, 12–14] .

In the blood stream, the majority of 25OHD and other VD sterols are bound to the VD binding protein (VDBP, encoded by the *GC* gene) [15–18], which was initially named as group-specific protein [16]. In healthy people, the circulating VDBP concentration seems to be independent of genetic or racial differences [19], age, adiposity [20], sunshine exposure [21], concentrations of VD sterols, and other hormones [22]; however, both 25OHD and VDBP are predominantly synthesized and secreted by the liver, and their circulating concentrations are impaired by hepatic disease [23, 24]. Moreover, sunshine exposure (including season, latitude, exposure time of the day, and so on), skin colour, VD-rich food, and others affect the circulating concentration of 25OHD. It is also intricately affected by its affinity to VDBP, which is determined by the isoforms of VDBP or the polymorphisms of *GC*. Of the identified more than 120 single nucleotide polymorphisms (SNPs), rs7041 and rs4588 are the most commonly concerned because they are missense SNPs located on the 11th exon of *GC*. Rs7041T  > G encodes aspartic acid  > glutamic acid, and rs4588C > A encodes threonine > lysine. Combined with the alleles of the two SNPs in complete linkage disequilibrium [25], *GC* has three high-frequency allelic variants. The most frequent variant is designated as *GC*1f (rs7041T-rs4588C); rs7041G defines the *GC*1s form (rs7041G-rs4588C), and rs4588A defines *GC*2 (rs7041T-rs4588A) [26]. Thus, the *GC* gene has 6 genotypes: 1f/1f, 1s/1s, 1f/1s, 2/2, 1f/2, and 1s/2. If *GC*1f and *GC*1s are combined, then *GC*1/1 is equal to rs4588C/C, *GC*1/2 is equal to rs4588C/A, and *GC*2/2 is equal to rs4588A/A in genotypic frequencies.

Although studies have suggested that the *GC*2 haplotype has lower circulating concentrations of VDBP and 25OHD in certain races or populations [27–29], others have challenged these findings by not finding the affinity difference [30, 31] or even the opposite relationship [32]. The present study analyses the effect of the *GC* genotype on the plasma 25OHD concentration in a Han Chinese population living in South China, illuminates the concern about the genotypes of *GC* when using 25OHD to evaluate the VD status, and explores the association between *GC* genotypes and specific phenotypes associated with VD deficiency.

## Methods

### Participants and sample collection

This study was approved by the Ethic Committee of Shenzhen Centre of Chronic Disease Control and had the registration ID of NCT03406234 in the ClinicalTrials.gov online system. Participants, randomly recruited over one year at a health examination centre in Shenzhen City, were informed of the purpose of the questionnaire, physical examination, and blood sampling, and they provided their written consent. Health history and food intake data were collected via face-to-face interviews using a computerized version of the questionnaire applied in China’s 2010 Chronic Disease and Risk Factor Surveillance [33], into which questions regarding sunshine exposure and sun protection use (collected by 3 categorized values: 1 = never use; 2 = use always under sunshine; and 3 = use only under strong sunlight) were incorporated. VD intakes were calculated using the dietary VD concentration data published by the US Department of Agriculture (USDA Nutrient Database for Standard Reference, Release 28. Nutrient Data Laboratory Home Page, 2018) and the VD supplement dosage if VD supplementations were used by some participants.

The inclusion criteria for the participants were ≥ 18 years old, member of the Han population, living in Shenzhen for > 2 years, not pregnant, and free of hepatic and renal disease. Participants were grouped as being VD deficient (VDD), VD insufficient (VDI), or VD sufficient (VDS) with plasma 25OHD concentrations of < 50, 50 - < 75, or ≥ 75 nmol/L, respectively [2, 13, 14, 34].

Overnight (> 10 h) fasting blood samples were drawn from the ulnar vein and collected with a 10-mL EDTA-K_2_ anticoagulate tube (Cat. #: 366643, BD Company). Concentrations of red blood cells and haemoglobin were determined with whole blood samples. The samples of plasma and blood cells were prepared via centrifugation at 4 °C 3000×g for 10 min.

### Biochemical assays

The concentrations of fasting plasma glucose (FPG), high-density lipoprotein cholesterol (HDLC), low-density lipoprotein cholesterol (LDLC), total cholesterol, and triglycerides in fresh plasma were determined with a Beckman-LX20 automatic analyser. Red blood cells and haemoglobin were analysed with an XS-800i haematology analyser (Sysmex Corporation, Shanghai, China). A plasma concentration of 25OHD was analysed with the enzyme-linked immunosorbent assay (ELISA) method (Cat. #: AC-57F1, IDS Ltd., UK) validated by the Vitamin D External Quality Assessment Scheme as previously described [34, 35]. The VDBP concentration was measured using a commercial ELISA kit (Cat. #: LE-H5018, Lai Er Bio-Tech, Hefei, China) on a Multiskan Go microplate reader (Thermo-Fisher Scientific Inc.).

### Genotype analysis

DNA samples were prepared from the blood cells with a commercial kit (Cat. #: D3392–02, Omega Bio-tek) for genotyping using the molecular beacon method on a Roche 480II qPCR validated by sequencing (Thermo-Fisher, Shanghai, China). Briefly, a pair of primers and a molecular beacon probe were designed to determine the genotypes of the two adjacent SNPs of rs7041 and rs4588, and the asymmetric qPCR program was established for a melting curve analysis [36]. The forward primer sequence was 5’-GGTTTTTCAGACTGGCAGAGCGACTA-3′, the backward primer sequence was 5’-GAGGTGAGTTTATGGAACAGCA-3′, and the molecular beacon probe was 5′-fluorophore-cacagCTGA**T**GCCACACCCA**A**GGAACTGtg-quencher-3′ (bold underlined bases were the alleles for rs7041 and rs4588, respectively). The genotypes were determined using the different melting curves (Additional file 1: Figure S1).

### Statistical analyses

A one-way ANOVA followed by Bonferroni test was performed for the numerical parameters, and χ^2^-tests were performed for the percentage or frequency data using SPSS19.0 (IBM). The genotypic frequencies across participants was examined using the Hardy-Weinberg equilibrium (HWE) for sampling representativeness. The contribution of different genotypes to VD status were analysed with logistic regressions. Differences with statistical probabilities less than 0.05 (*P* <  0.05) were accepted as significant.

## Results

### The clinical profiles and biochemical measures of participants

A total of 2641 participants (1119 males and 1522 females) were included in this study. The clinical profiles are summarized in Table 1. More than half of the participants showed a VDI status, (i.e., 61.4% of males and 58.0% of females), and the combined rates of VDD and VDI were 80.2% in males and 86.1% in females. The rate of VD supplementation use (primarily fish oil) was less than 10%, and the VD supplement dosages for those participants were included for total VD intake adjustment in the logistic analysis. No participant showed a potentially toxic VD status (plasma 25OHD > 250 nmol/L), and the highest level was 165 nmol/L.

**Table 1:** Clinic profiles of subjects at vitamin D (VD) deficient (VDD), VD insufficient (VDI), and VD sufficient (VDS) statuses ^1^

Parameters ^2^	Males, *n* = 1119			Overall *P*-value	Female, *n* = 1522			Overall *P*-value
	VDD	VDI	VDS		VDD	VDI	VDS	
Plasma 25OHD, nmol/L	42.9 ± 5.7 ^a^	62.0 ± 6.8 ^b^	86.0 ± 11.0 ^c^	**< 0.001**	41.7 ± 6.4 ^a^	61.0 ± 6.6 ^b^	85.8 ± 11.7 ^c^	**< 0.001**
VD status, *n* (%)	210 (18.8%)	687 (61.4%)	222 (19.8%)		428 (28.1%)	883 (58.0%)	211 (13.9%)	
VD supplement user, *n* (%)	18 (8.6%)	46 (6.7%)	14 (6.3%)		25 (5.8%)	55 (6.2%)	23 (10.9%)	
Sunshine exposure, min/d	34.7 ± 36.7	38.6 ± 43.0	41.5 ± 45.0	0.25	31.2 ± 31.2	30.9 ± 35.1	33.5 ± 37.5	0.63
Age, y	37.0 ± 10.6	37.4 ± 10.2	38.0 ± 10.5	0.64	37.6 ± 11.1 ^a^	39.7 ± 12.2 ^b^	43.2 ± 13.5 ^c^	**< 0.001**
Body mass index, kg/m^2^	24.7 ± 3.2	24.4 ± 2.8	24.2 ± 2.9	0.23	21.7 ± 2.8 ^a^	22.1 ± 2.8 ^b^	22.0 ± 2.5 ^b^	**0.02**
Total VD intake, IU/d	132 ± 76	129 ± 71	136 ± 84	0.51	119 ± 71	123 ± 65	127 ± 64	0.31
From staple food	58.7 ± 35.8	56.7 ± 33.7	57.0 ± 34.1	0.75	50.0 ± 33.8	53.4 ± 33.2	53.9 ± 31.8	0.19
From meat and fish ^3^	14.9 ± 13.5	14.6 ± 12.4	14.4 ± 11.1	0.89	12.0 ± 8.7 ^a^	13.8 ± 10.4 ^b^	13.4 ± 8.9 ^a,b^	**0.007**
From egg and milk	48.9 ± 51.7	50.0 ± 51.4	57.0 ± 65.2	0.20	49.6 ± 52.5	48.3 ± 46.2	47.5 ± 40.7	0.84
From mushroom	1.22 ± 1.61	1.30 ± 1.77	1.21 ± 1.45	0.70	1.11 ± 1.31	1.14 ± 1.36	1.13 ± 1.26	0.94
SBP, mmHg	125.7 ± 13.8	123.4 ± 14.6	123.8 ± 14.7	0.16	114.5 ± 15.7	115 ± 16.8	115.9 ± 15.3	0.59
DBP, mmHg	76.5 ± 10.0	75.0 ± 10.8	74.9 ± 9.9	0.18	67.6 ± 10.5	67.5 ± 10.2	69.1 ± 10.4	0.12
FPG, mmol/L	5.7 ± 1.4 ^a^	5.5 ± 0.9 ^b^	5.4 ± 0.8 ^b^	**0.02**	5.4 ± 0.7	5.4 ± 0.9	5.4 ± 0.7	0.80
HDLC, mmol/L	1.40 ± 0.21 ^a^	1.37 ± 0.21 ^a,b^	1.35 ± 0.22 ^b^	**0.03**	1.45 ± 0.22	1.46 ± 0.24	1.44 ± 0.25	0.32
LDLC, mmol/L	3.00 ± 0.55 ^a^	2.91 ± 0.53 ^a,b^	2.87 ± 0.54 ^b^	**0.03**	2.88 ± 0.61	2.88 ± 0.59	2.91 ± 0.62	0.85
Triglycerides, mmol/L	2.3 ± 2.3 ^a^	1.9 ± 1.6 ^b^	1.7 ± 1.9 ^b^	**0.002**	1.2 ± 1.5	1.2 ± 0.9	1.1 ± 0.8	0.65
Total cholesterol, mmol/L	5.2 ± 1.0	5.1 ± 0.9	5.1 ± 0.9	0.13	5.0 ± 1.0	5.1 ± 1.0	5.1 ± 1.1	0.45
Red blood cells, 10^12^/L	5.1 ± 0.4	5.1 ± 0.4	5.1 ± 0.4	0.86	4.4 ± 0.3	4.4 ± 0.4	4.5 ± 0.4	0.63
Hemoglobin, g/L	148.6 ± 10.2	148.2 ± 10.0	147.3 ± 9.2	0.37	126.9 ± 10.1	127.0 ± 10.3	127.7 ± 10.2	0.60
VD binding protein, mg/L	239.6 ± 26.7	238.9 ± 27.7	239.6 ± 28.4	0.91	237.9 ± 29.5	238.3 ± 27.5	236.7 ± 29.5	0.75

^1^VDD, VDI, and VDS were defined as plasma 25OHD concentrations of < 50, 50 - < 75, and ≥ 75 nmol/L, respectively. Data are means ± SD or *n* (%). In the post hoc comparison when the overall *P*-value is < 0.05, data differ without a same superscript letter, *P* < 0.05

^2^Abbreviations: *DBP* diastolic blood pressure, *FPG* fasting plasma glucose, *HDLC* plasma high-density lipoprotein cholesterol, *LDLC* plasma low-density lipoprotein cholesterol, *SBP* systolic blood pressure

^3^Including pork, beef, chicken, and fish

The self-reported mean times of sunshine exposure were 34.7–41.5 min for males and 30.9–33.5 min for females per day. Between different VD statuses, sunshine exposure time did not differ in both (*P* = 0.09) or either of the genders (*P* = 0.25 for males and 0.63 for females, Table 1), while males had higher sunshine exposure time than females in total (*P* <  0.001), VDI (*P* <  0.001), or VDS status (*P* = 0.04). The percentages of never using sun protection was 84% in males and 18.3% in females. The means of the total VD intakes were 129–136 IU/d for males and 119–127 IU/d for females, and these values did not significantly differ across the VD statuses of either gender (*P* = 0.51 for males and 0.31 for females). Males had higher total VD intakes (*P* = 0.001). No significant differences were found with regard to the VD intakes from staple food, egg and milk, and mushroom between the VD statuses of either gender; however, VDD females showed significant lower VD intakes from meat and fish than VDI females (*P* = 0.005). The mean plasma VDBP concentration was approximately 240 mg/L and did not differ across the VD nutritional subgroups (*P* = 0.91 for males and 0.75 for females, Table 1) or the *GC* genotypes (*P* = 0.45 for males and 0.11 for females, Additional file 1: Table S1).

In males, the circulating concentrations of FPG, HDLC, LDLC, and triglycerides were higher in group(s) with lower VD nutritional statuses than the group(s) with higher VD nutritional status (*P* <  0.05). In female participants, however, no differences were found in the assayed biochemical parameters amongst the VD groups, and a higher VD status was associated with an older age (*P* <  0.001). VDD females also had lower body mass indices (BMIs) than those with a higher VD status (*P* = 0.02, Table 1). When the FPG and lipid profiles of males were compared between the subgroups divided by the genotypes of rs4588, no significant differences were identified (*P* > 0.05, Additional file 1: Table S2).

In the partial correlation analyses between the plasma 25OHD concentration and its affecting factors, 25OHD was not significantly correlated with BMI (*P* = 0.14), sunshine exposure time (*P* = 0.09), or the plasma VDBP concentration (*P* = 0.39); however, it was positively correlated with age (*P* <  0.01, Additional file 1: Table S3).

### Genotypes of *GC* and its effect on VD status

**Fig. 1:**
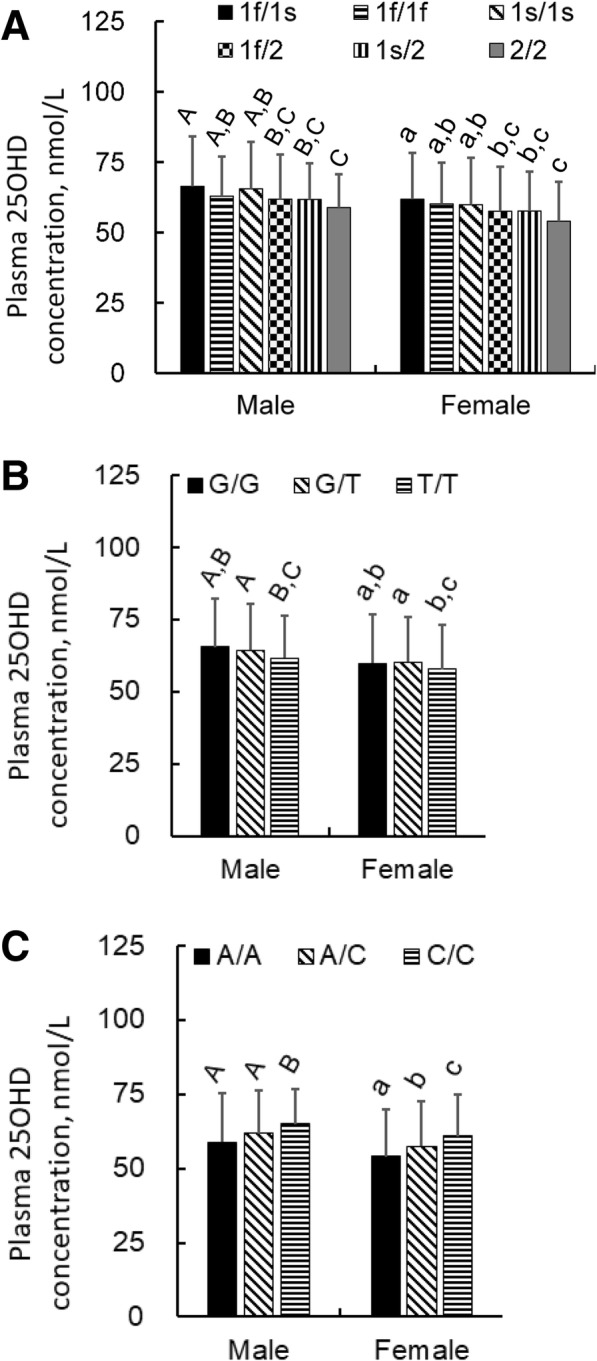
Plasma 25OHD concentrations in males and females with genotypes of rs7041-rs4588 (**a**), rs7041 (**b**), and rs4588 (**c**) in vitamin D binding protein gene. Data are means ± SD, and differ without a common letter, *P* < 0.05. For the 6 genotypes of 1f/1f, 1s/1s, 1f/1s, 2/2, 1f/2, and 1s/2, *n* = 212, 98, 261, 88, 278, and 182 in males and 253, 116, 405, 140, 356, and 252 in females, respectively. For the G/G, G/T, and T/T genotypes of rs7041, *n* = 98, 443, and 578 in males and 116, 657, and 749 in females, respectively. For the A/A, A/C, and C/C genotypes of rs4588 (or *GC*2/2, *GC*1/2, and *GC*1/1 of VDBP), *n* = 88, 460, and 571 in males and 140, 608, and 774 in females, respectively

Both of the minor allele frequencies (MAFs) of rs7041G and rs4588A were 0.289 in the total population. The genotypic and allelic frequencies of rs7041-rs4588, rs7041, and rs4588 are summarized in Table 2. Except for the genotypic frequency of rs7041 in males, all of the frequencies in both males and females differed across VD statuses (*P* <  0.05). The allelic frequency rank of rs7041G was VDD < VDI < VDS, whereas that of rs4588A was reversed in both males and females. Figure 1 compares the plasma 25OHD concentrations in the subgroups of different genotypes. In both genders, 1f/1s showed significantly higher plasma 25OHD concentrations than all three rs7041T-rs4588A (*GC*2) haplotype carriers (*GC*2/2, *GC*1f/2, and *GC*1s/2. *P* = 0.001, 0.008, and 0.026 respectively for males and <  0.001, 0.002, and 0.011 respectively for females). Significantly lower plasma 25OHD concentrations were observed in the rs7041T/T homozygotes than the rs7041G/T heterozygotes (*P* = 0.016 for males and 0.009 for females) but not the rs7041G/G homozygotes (*P* = 0.08 for males and 0.53 for females). Conversely, the rs4588A/A homozygotes had lower plasma 25OHD concentrations than the rs4588C/C homozygotes (*P* = 0.002 for males and < 0.001 for females).

**Table 2:** Univariate analyses of the genotypic or allelic frequencies of rs7041 and rs4588 in vitamin D binding protein gene^1^

Genotypes or alleles^2^	Male, *n* = 1119				Female, *n* = 1522			
	VDD, % *n* = 210	VDI, % *n* = 687	VDS, % *n* = 222	Overall *P*-value	VDD, % *n* = 428	VDI, % *n* = 883	VDS, % *n* = 211	Overall *P*-value
rs7041-rs4588								
1f/1s	19.0	23.0	28.4	0.004	20.8	27.5	34.6	< 0.001
1f/1f	17.1	20.1	17.1		14.3	17.3	18.5	
1s/1s	6.7	8.3	12.2		7.5	7.0	10.4	
2/2	9.0	9.3	2.3		13.1	8.4	4.7	
1f/2	31.4	22.7	25.2		26.9	23.3	16.6	
1s/2	16.7	16.6	14.9		17.5	16.4	15.2	
rs7041								
G/G	6.7	8.3	12.2	0.054	7.5	7.0	10.4	0.011
G/T	35.7	39.6	43.2		38.3	43.9	49.8	
T/T	57.6	52.1	44.6		54.2	49.0	39.8	
G	24.5	28.1	33.8	0.009	26.6	29.0	35.3	0.006
T	75.5	71.9	66.2		73.4	71.0	64.7	
rs4588								
A/A	9.0	9.3	2.3	0.001	13.1	8.4	4.7	< 0.001
A/C	48.1	39.3	40.1		44.4	39.8	31.8	
C/C	42.9	51.4	57.7		42.5	51.9	63.5	
A	33.1	29.0	22.3	0.002	35.3	28.3	20.6	< 0.001
C	66.9	71.0	77.7		64.7	71.7	79.4	

^1^ Subjects were defined as vitamin D (VD) deficient (VDD), VD insufficient (VDI), and VD sufficient (VDS) statuses, having plasma 25OHD concentration < 50, 50 - < 75, and ≥ 75 nmol/L, respectively

^2^ Haplotype of rs7041-rs4588: 1f, T-C; 1s, G-C; 2, T-A

**Fig. 2:**
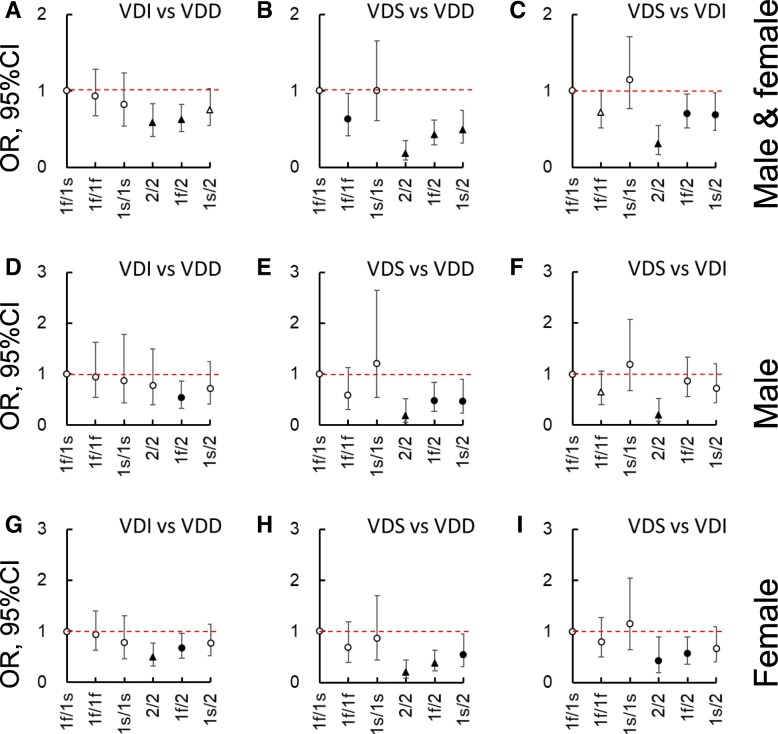
Logistic analysis on the contribution of vitamin D binding protein genotypes (1f/1f, 1 s/1 s, 2/2, 1f/2, or 1 s/2 against 1f/1 s) to the vitamin D (VD) statuses after adjusting for (gender,) age, body mass index, sampling month, smoking status, sunshine exposure time, sun protection use, and total vitamin D intakes. **a**, **d**, and **g** are VD insufficient (VDI) vs VD deficient (VDD), **b**, **e**, and **h** are VD sufficient (VDS) vs VDD, and **c**, **f**, and **i** are VDS vs VDI in both genders, male, and female, respectively. Data are odds ratio (OR, represented by ○, △, ●, and▲) ± 95% of confidential interval (CI). ○, *P* ≥ 0.1; △, 0.05 ≤ *P* < 0.1; ●, 0.01 ≤ *P* < 0.05; ▲, *P* < 0.01

**Fig. 3:**
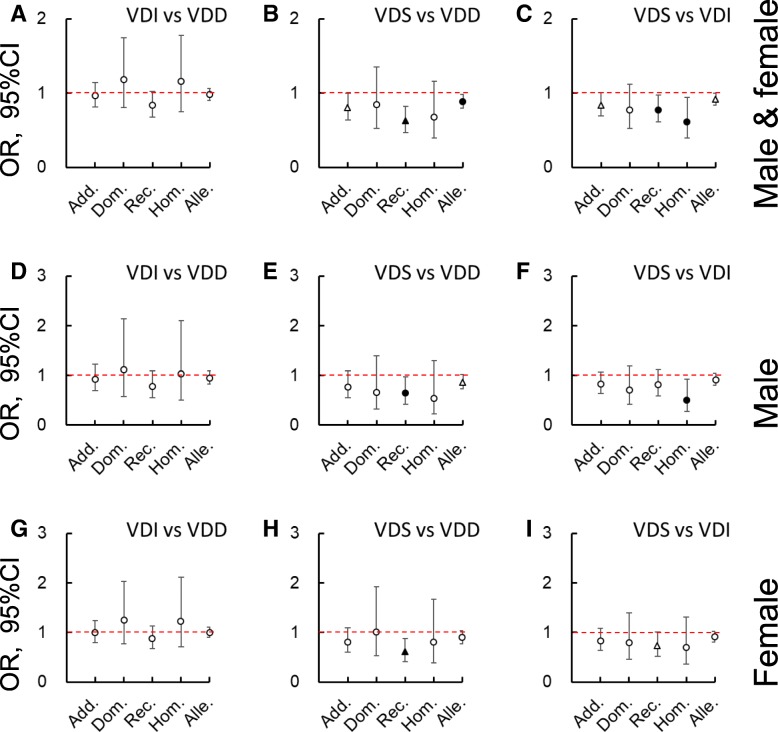
Logistic analysis on the contribution of rs7041 in different genotypic models to the vitamin D (VD) statuses after adjusting for (gender,) age, body mass index, sampling month, smoking status, sunshine exposure time, sun protection use, and total vitamin D intakes. **a**, **d**, and **g** are VD insufficient (VDI) vs VD deficient (VDD), **b**, **e**, and **h** are VD sufficient (VDS) vs VDD, and **c**, **f**, and **i** are VDS vs VDI in both genders, male, and female, respectively. Data are odds ratio (OR, represented by ○, △, ●, and▲) ± 95% of confidential interval (CI). ○, *P* ≥ 0.1; △, 0.05 ≤ *P* < 0.1; ●, 0.01 ≤ *P* < 0.05; ▲, *P* < 0.01. Add., additive model; Alle., allelic model; Dom., dominant model; Hom., homozygous model; Rec., recessive model

**Fig. 4:**
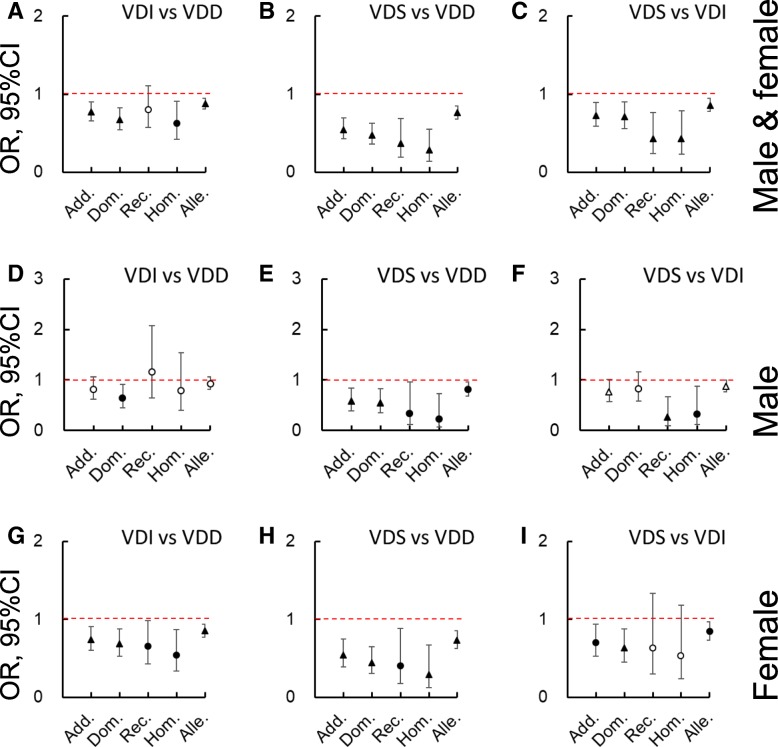
Logistic analysis on the contribution of rs4588 in different genotypic models to the vitamin D (VD) statuses after adjusting for (gender,) age, body mass index, sampling month, smoking status, sunshine exposure time, sun protection use, and total vitamin D intakes. **a**, **d**, and **g** are VD insufficient (VDI) vs VD deficient (VDD), **b**, **e**, and **h** are VD sufficient (VDS) vs VDD, and **c**, **f**, and **i** are VDS vs VDI in both genders, male, and female, respectively. Data are odds ratio (OR, represented by ○, △, ●, and▲) ± 95% of confidential interval (CI). ○, *P* ≥ 0.1; △, 0.05 ≤ *P* < 0.1; ●, 0.01 ≤ *P* < 0.05; ▲, *P* < 0.01. Add., additive model; Alle., allelic model; Dom., dominant model; Hom., homozygous model; Rec., recessive model

Logistic analyses were performed to compare the contribution of the different *GC* genotypes to VD status after adjusting for (gender,) age, BMI, sampling month, smoking status, sunshine exposure, sun protection use, and total VD intakes. Figures 2, 3 and 4 were created using the odds ratio (OR) and 95% confidential interval (CI) data. Taking the 1f/1s genotype as the reference, 2/2, 1f/2, and/or 1s/2 significantly decreased the possibility of higher VD status (*P* < 0.05). Namely, it increased the risk of lower VD status. Furthermore, when the two SNPs were separately examined with additive, dominant, recessive, homozygous, and allelic models, a negative contribution was more frequently found for rs4588 than rs7041 when comparing the higher with the lower statuses (i.e., VDI vs VDD, VDS vs VDD, or VDS vs VDI, *P* < 0.05), especially amongst females.

## Discussion

### The effect of VDBP on the 25OHD concentration

No differences in the VDBP concentrations were found using polyclonal antibody method [37] or the LC-MS/MS method [19, 38], though the ELISA method with a monoclonal antibody for VDBP may induce bias [38–42]. Using an ELISA kit with a polyclonal antibody, we did not find a difference in the plasma VDBP concentrations between the subgroups of participants (Table 1 and Additional file 1: Table S1), nor was the VDBP concentration correlated with the plasma 25OHD concentration (Additional file 1: Table S3).

Although the varied 25OHD concentrations might not result from the constant concentration of VDBP in healthy people [20, 43], the missense variations in rs7041 and rs4588 might affect the affinity of VDBP to VD sterols. The rs4588A base pair leading to the lysine amino acid in the *GC*2 protein lacked the preferred site of O-linked glycosylation, which might reduce the affinity of VDBP to VD sterols, and result in a lower 25OHD concentration in many populations [28, 40, 44–55]. While rs7041 was tightly linked with rs4588 on the same exon, and thus both of the two SNPs are associated with 25OHD concentrations [56]. An additional copy of rs7041T or rs4588A was associated with lower 25OHD concentrations in premenopausal white women [57]. Rs7041T/T, rs4588A/A, *GC*1f/2, and *GC*2/2 were associated with lower 25OHD levels in a cross-sectional survey of healthy girls from southern Brazil [58]. In a rural region of the Gambia in western Africa, the plasma 25OHD concentration was higher in homozygotes for *GC*1f compared with other *GC* variants [59]. Lin’s group also found that rs4588A and rs7041T had lower plasma 25OHD concentration in adult Chinese Hans living in high (Beijing) and mid (Shanghai) latitude cities [60]; and further, rs4588C had higher 25OHD increase by oral VD supplementation [61]. However, an inconsistency was observe in certain studies [28, 32, 49, 51, 54], which might be explained by the fact that rs7041T exists in both *GC*1f and *GC*2, which have the highest and lowest affinities to 25OHD, respectively [46], and presented a splitting effect between the two opposite phenotypes in certain instances. Moreover, a recent study found that a lower 25OHD level was associated with rs7041T/T but not with the genotypes of rs4588 [62].

Consistent with most findings, *GC*2/2 had the lowest VD status in both male and female Han Chinese adults of the present study, and *GC*2 carriers (*GC*2/2, *GC*1f/2, and *GC*1s/2) had lower 25OHD values than *GC*1f/1s individuals, though they resided in much lower latitude. The plasma 25OHD concentration (nmol/L) means were 64.9, 61.8, and 58.9 in males and 61.0, 57.6, and 54.0 in females for the *GC*1/1 (rs4588C/C), *GC*1/2 (rs4588C/A), and *GC*2/2 (rs4588A/A) genotypes, respectively. It highly suggested a dose-response relationship between the *GC*2 haplotype (or rs4588A) copies and low VD status. The rs7041T/T subgroup showed a lower 25OHD than the rs7041G/T but not the rs7041G/G subgroup, having no suggestion for a dose-response relationship. The negative association of *GC*2 haplotype with VD status was confirmed by logistic regression analysis, and rs4588A was more precise or sensitive than rs7041T to predict the risk of low VD nutritional status in both or either gender(s).

### Other factors influencing VD status

Excluding the contribution of genetic differences, the 25OHD levels in individuals not taking supplements largely depend on sunshine exposure, diet [52, 63], and indirect factors that affect the bioavailability or synthesis of VD such as age, BMI, gender, and so on. Although the sunshine exposure means amongst our male participants seemed to increase with VD status (Table 1), the great variation in this sample did not support a significant difference, and this result was not correlated with plasma 25OHD concertation (Additional file 1: Table S3). This explanation might be a limitation of the method used to collect sunshine exposure data. Similarly, dietary VD intakes also seemed to have limited effects on the VD status amongst the subgroups. The lower VD intakes from meat and fish in VDD females suggest an intentional bias in food choice to keep slim, whereas VDS females might be more likely to benefit from the VD supplements consumption.

The overall concentration of plasma 25OHD in females was much lower than that in males (*P* < 0.001), and the means of age increased with VD status in females (*P* < 0.001, Table 1). These findings are consistent with those of previous studies [64–66]. These effects might be explained by less outdoor activity of labour ages and sunshade habits including the use of sunscreen, sun hats, and parasols, especially amongst young women [57, 65].

Because VD sterols are fat soluble, obesity might reduce the circulating 25OHD concentration, and negative associations between BMI and 25OHD have been reported by many studies [67, 68]. In certain situations [35, 67, 69, 70], including a Chinese national report [66] and the present study, however, a null association has been found between these two parameters, most likely because BMI is not a perfect marker for obesity, especially the lower BMI range with regard to the Han Chinese population.

### VD nutritional status and biochemical profiles

Lower VD status was coupled with higher FPG, HDLC, LDLC, and triglyceride levels in the current male participants (Table 1) as well as coupled with an increasing number of rs4588A genotypes (Additional file 1: Table S2). However, the FPG and lipid levels did not differ across the rs4588 genotypes. Thus, VD status and not the genetic variations of VDBP were associated with these parameters in males.

To explore the cause and effect between VD status and ill health, a recent meta-analysis confirmed the moderate-to-strong inverse associations between 25OHD concentrations and various outcomes of observational studies, including serum lipid concentrations and glucose metabolism disorders [3]. However, the results from intervention studies have not supported the benefit of VD supplementation to reverse these disorders [3], which implies that low 25OHD is a marker of ill health and not the cause. If so, then our data suggest that males are more likely to have glucose and lipid metabolic disorders to affect their VD status than females.

### Limitations

In the present study, the sample size of the populations was moderate, and the measured 25OHD concentration was for the total instead of free and bioavailable fractions, which were suggested to be more accurate for revealing the relevance of 25OHD with the health outcomes [15, 71, 72]. These limitations might lead to the null associations of circulating 25OHD concentration with FPG and lipid profiles.

## Conclusions

The *GC*2 haplotype of VDBP is a risk factor for a lower circulating 25OHD concentration in an adult Han population in southern China, and rs4588 was more sensitive than rs7041 to predict the VD status. However, the *GC*2 haplotype was not related to FPG, HDLC, LDLC, or triglyceride levels in either gender, though these glucose/lipid profiles were higher in males with lower VD status.

## Additional file

Additional file 1: **Table S1.** Plasma concentration of vitamin D binding protein at its different genotypes. **Table S2.** Metabolic parameters regarding glucose and lipids in male subjects grouped by the genotypes of vitamin D binding protein gene. **Table S3.** Partial correlation analysis on 25OHD and interested variants. **Figure S1.** Typical genotyping graph of 6 genotypes in vitamin D binding protein gene analyzed by molecular beacon probe-based qPCR method. (DOCX 108 kb)

## Abbreviations

25OHD: 25-hydroxyvitamin D
BMI: Body mass index
CI: Confidential interval
DBP: Diastolic blood pressure
ELISA: Enzyme-linked immunosorbent assay
FPG: Fasting plasma glucose
HDLC: High-density lipoprotein cholesterol
HWE: Hardy-Weinberg equilibrium
LDLC: Low-density lipoprotein cholesterol
MAF: Minor allele frequency
OR: Odds ratio
SBP: Systolic blood pressure
VD: Vitamin D
VDBP: VD binding protein
VDD: VD deficient
VDI: VD insufficient
VDS: VD sufficient
