# Supplementary material for: The GC2 haplotype of the vitamin D binding protein is a risk factor for a low plasma 25-hydroxyvitamin D concentration in a Han Chinese population
Source: Nutr Metab (Lond). 2019 Jan 14;16:5. doi: 10.1186/s12986-019-0332-0 (PMC6332541; doi:10.1186/s12986-019-0332-0)
Supplement: Supplementary file 1 — Table S1. Plasma concentration of vitamin D binding protein at its different genotypes. Table S2. Metabolic parameters regarding glucose and lipids in male subjects grouped by the genotypes of vitamin D binding protein gene. Table S3. Partial correlation analysis on 25OHD and interested variants. Figure S1. Typical genotyping graph of 6 genotypes in vitamin D binding protein gene analyzed by molecular beacon probe-based qPCR method. (DOCX 108 kb) [file 12986_2019_332_MOESM1_ESM.docx]

**Additional file 1**

**Additional file 1:Table S1.** Plasma concentration of vitamin D binding protein at its different genotypes, mg/L

|  | Total, *P* = 0.06 | |  | Male, *P* = 0.45 | |  | Female, *P* = 0.11 | |
| --- | --- | --- | --- | --- | --- | --- | --- | --- |
| Genotypes | Mean ± SD | *n* |  | Mean ± SD | *n* |  | Mean ± SD | *n* |
| 1s/1s | 239.0 ± 29.8 | 465 |  | 239.3 ± 28.7 | 212 |  | 238.7 ± 30.8 | 253 |
| 1f/1f | 241.3 ± 30.0 | 214 |  | 243.7 ± 29.8 | 98 |  | 239.3 ± 30.1 | 116 |
| 2/2 | 236.5 ± 26.0 | 228 |  | 239.2 ± 26.0 | 88 |  | 234.8 ± 26.0 | 140 |
| 1s/1f | 240.5 ± 28.6 | 666 |  | 239.8 ± 28.9 | 261 |  | 240.9 ± 28.3 | 405 |
| 1s/2 | 237.2 ± 27.3 | 434 |  | 239.1 ± 25.9 | 182 |  | 235.8 ± 28.2 | 252 |
| 1f/2 | 236.6 ± 26.5 | 634 |  | 236.9 ± 26.3 | 278 |  | 236.4 ± 26.8 | 356 |
| Total | 238.5 ± 28.0 | 2641 |  | 239.2 ± 27.6 | 1119 |  | 238.0 ± 28.3 | 1522 |

**Supplementary data**

**Additional file 1: Table S2.** Metabolic parameters regarding glucose and lipids in male subjects grouped by the genotypes of vitamin D binding protein gene ^1^

| Parameters ^2^ | rs4588C/C  (*GC*1/1) | rs4588C/A  (*GC*1/2) | rs4588A/A  (*GC*2/2) | Overall *P*-value |
| --- | --- | --- | --- | --- |
| *n* | 571 | 460 | 88 |  |
| Plasma 25OHD, nmol/L | 64.9 ± 16.3 ^a^ | 61.8 ± 14.7 ^b^ | 58.9 ± 11.8 ^b^ | < 0.01 |
| Body mass index, kg/m^2^ | 24.5 ± 2.9 | 24.2 ± 2.9 | 24.5 ± 3.1 | 0.26 |
| FPG, mmol/L | 5.49 ± 0.87 | 5.55 ± 1.18 | 5.41 ± 1.02 | 0.37 |
| HDLC, mmol/L | 1.38 ± 0.21 | 1.37 ± 0.21 | 1.37 ± 0.22 | 0.84 |
| LDLC, mmol/L | 2.93 ± 0.54 | 2.92 ± 0.54 | 2.84 ± 0.55 | 0.37 |
| Triglycerides, mmol/L | 1.85 ± 1.53 | 1.94 ± 1.91 | 2.11 ± 3.02 | 0.42 |
| Total cholesterol, mmol/L | 5.13 ± 0.91 | 5.08 ± 0.91 | 5.06 ± 0.89 | 0.60 |

^1^ Data are means ± SD, and differ with different superscript letters, *P* < 0.05.

^2^ Abbreviations: 25OHD, 25-hydroxyvitamin D; FPG, fasting plasma glucose; HDLC, plasma high-density lipoprotein cholesterol; LDLC, plasma low-density lipoprotein cholesterol.

**Supplementary data**

**Additional file 1: Table S3.** Partial correlation analysis on 25OHD and interested variants ^1^

| Variant 1 | Variant 2 | *P*-value | Correlation | Adjusted factors |
| --- | --- | --- | --- | --- |
| 25OHD | Age | < 0.01 | 0.08 | BMI, education, gender, sampling month, smoking status, sunscreen usage, sunshine exposure time, and total VD intakes |
| 25OHD | BMI | 0.14 | -0.03 | age, education, gender, sampling month, smoking status, sunscreen usage, sunshine exposure time, and total VD intakes |
| 25OHD | Sunshine exposure time | 0.09 | 0.03 | age, BMI, education, gender, sampling month, smoking status, sunscreen usage, and total VD intakes |
| 25OHD | VDBP | 0.39 | -0.017 | age, BMI, education, gender, sampling month, smoking status, sunscreen usage, sunshine exposure time, and total VD intakes |

^1^ Abbreviations: 25OHD, 25-hydroxyvitamin D; BMI, body mass index; VD, vitamin D; VDBP, vitamin D binding protein.

**Supplementary data**

A


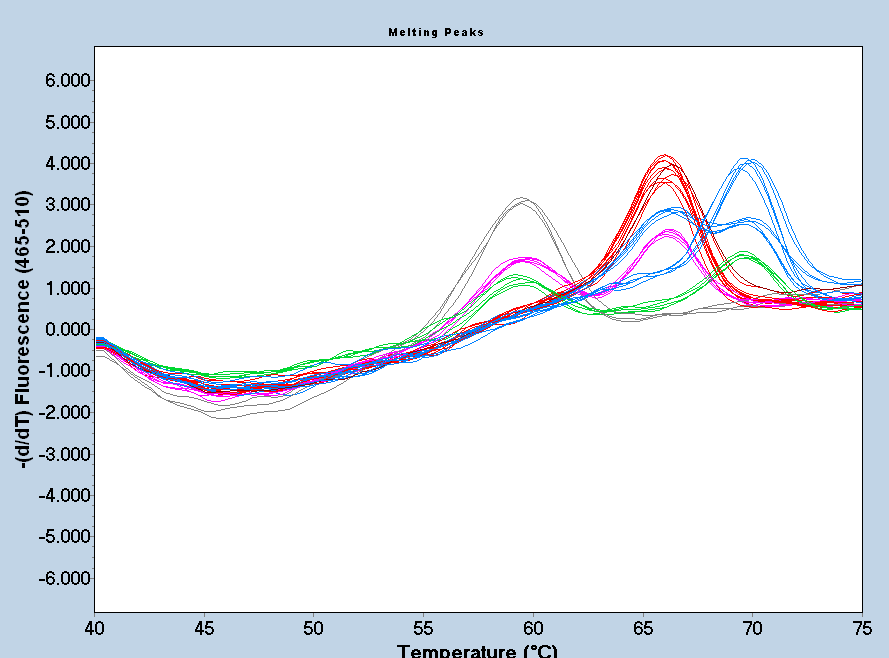


**Temperature (°C)**

**Melting Peaks**

B


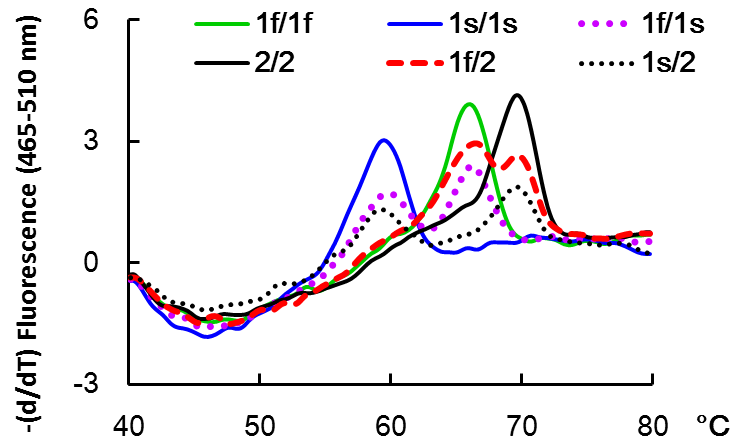


**Additional file 1: Figure S1.** Typical genotyping graph of 6 genotypes in vitamin D binding protein gene analyzed by molecular beacon probe-based qPCR method. For the samples tested on a 96-well plate, the melting curves generated by the Roche 480II software (LC480II SW1.5) **(A**) and the typical curves generated by Excel using the exported data (**B**) were illustrated.
